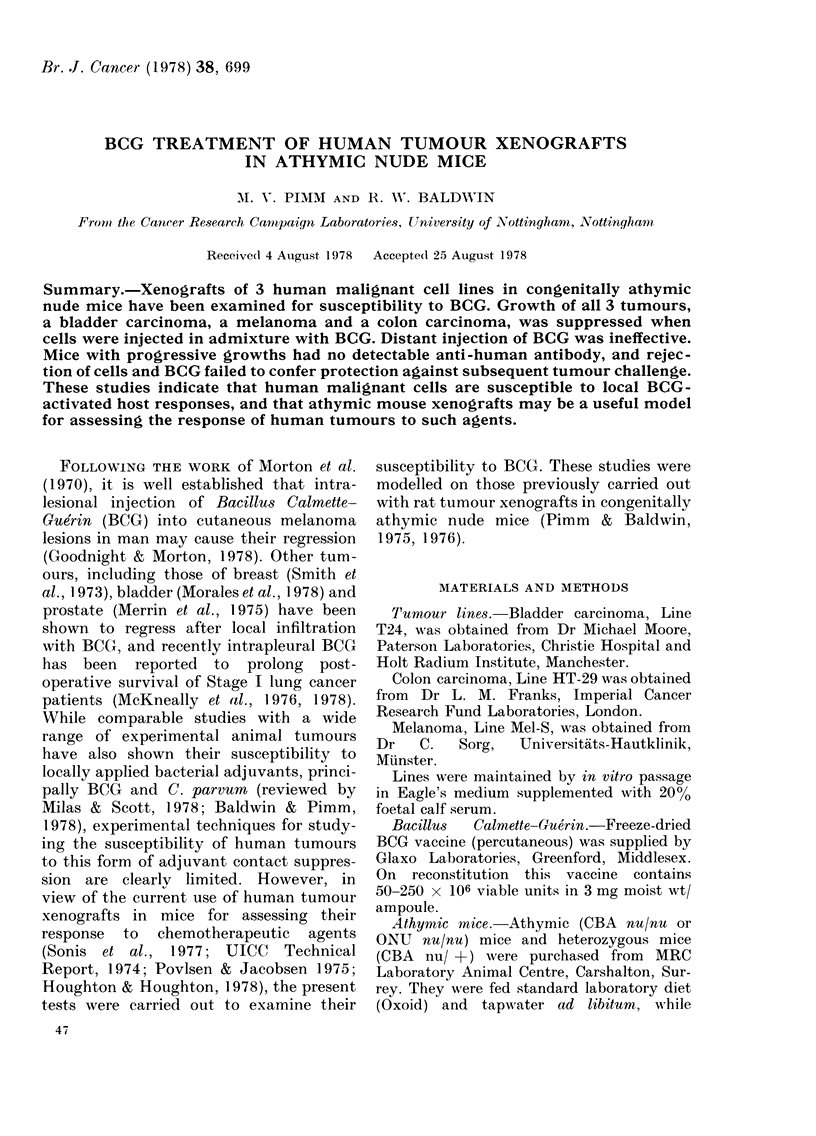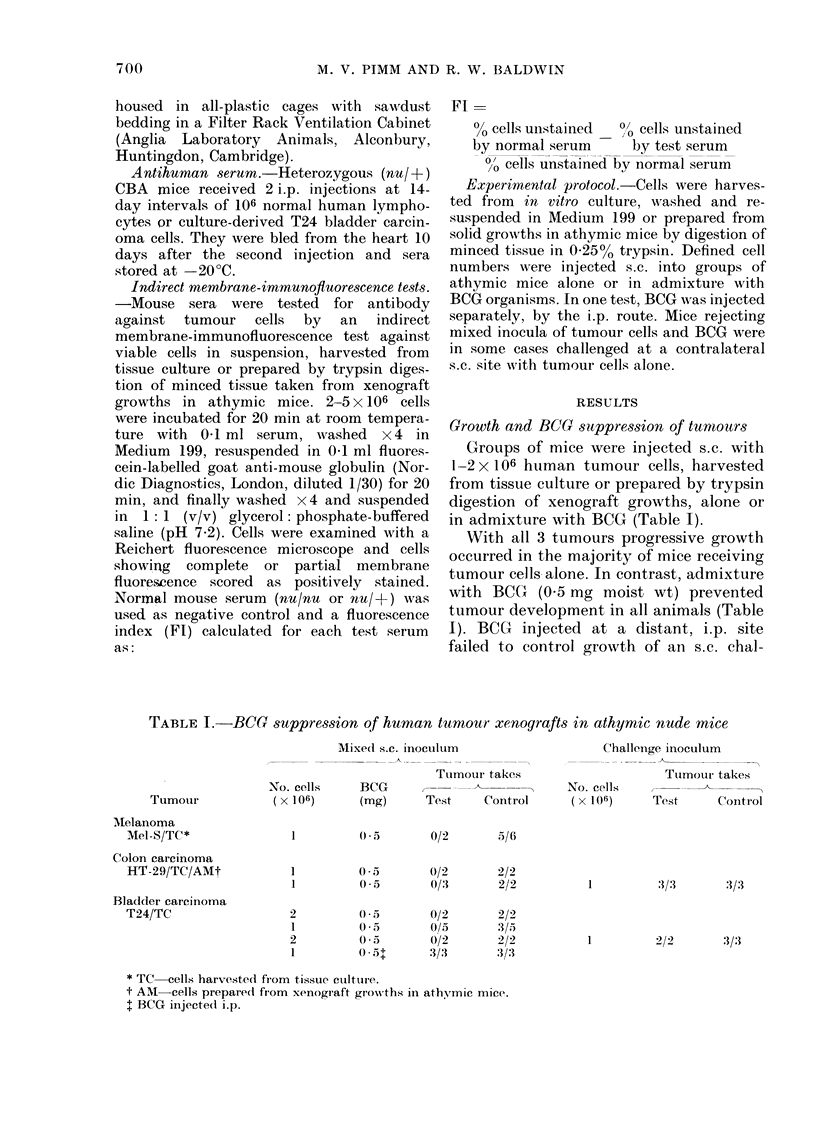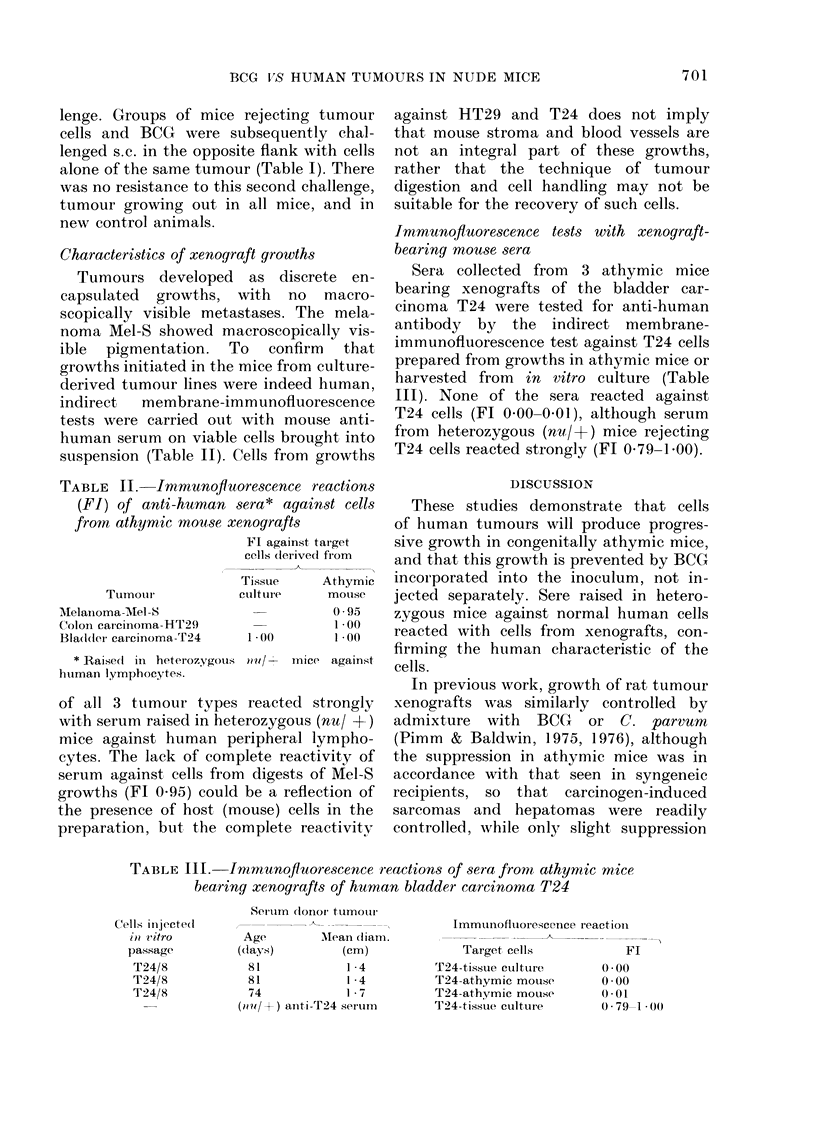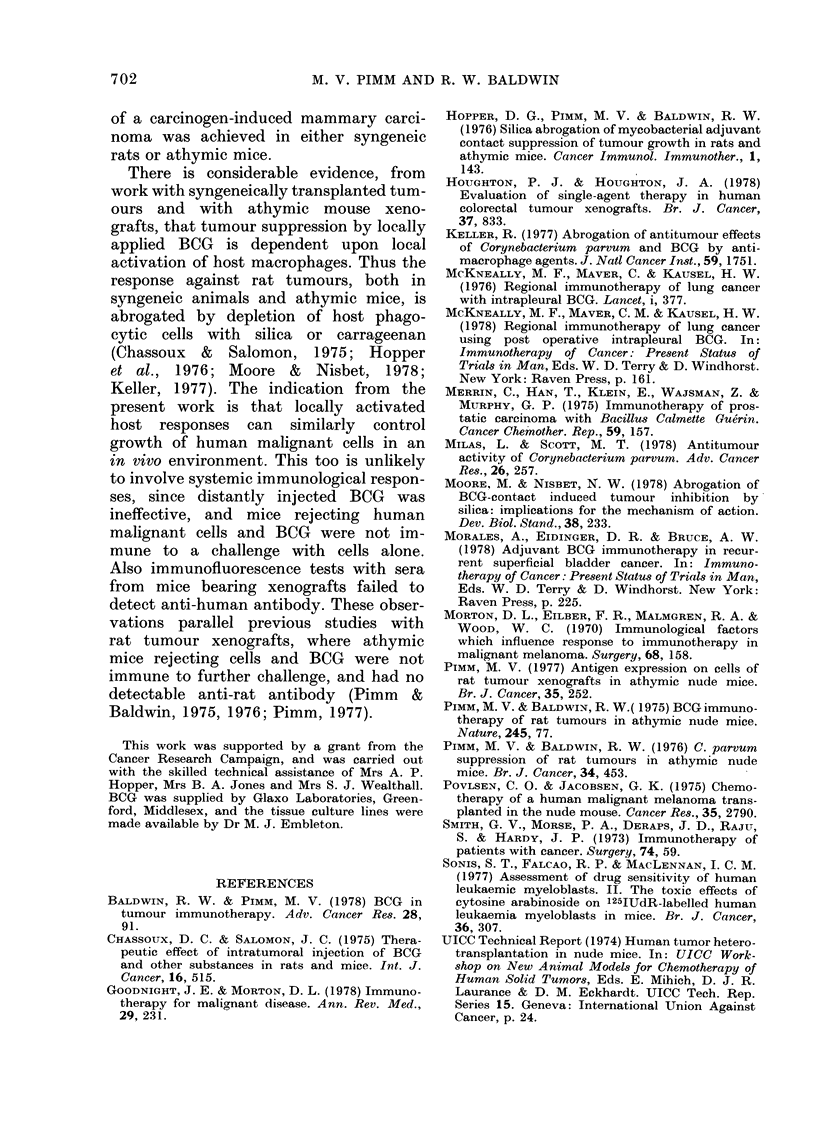# BCG treatment of human tumour xenografts in athymic nude mice.

**DOI:** 10.1038/bjc.1978.275

**Published:** 1978-12

**Authors:** M. V. Pimm, R. W. Baldwin

## Abstract

Xenografts of 3 human malignant cell lines in congenitally athymic nude mice have been examined for susceptibility to BCG. Growth of all 3 tumours, a bladder carcinoma, a melanoma and a colon carcinoma, was suppressed when cells were injected in admixture with BCG. Distant injection of BCG was ineffective. Mice with progressive growths had no detectable anti-human antibody, and rejection of cells and BCG failed to confer protection against subsequent tumour challenge. These studies indicate that human malignant cells are susceptible to local BCG-activated host responses, and that athymic mouse xenografts may be a useful model for assessing the response of human tumours to such agents.


*Br. J. Cancer* (1978) **38**, 699

### BCG TREATMENT OF HUMAN TUMOUR XENOGRAFTS IN ATHYMIC NUDE MICE

M. V. PIMM AND R. W. BALDWIN

*From the Cancer Research Campaign Laboratories, University of Nottingham, Nottingham*

Received 4 August 1978 Accepted 25 August 1978

**Summary.—Xenografts of 3 human malignant cell lines in congenitally athymic nude mice have been examined for susceptibility to BCG. Growth of all 3 tumours, a bladder carcinoma, a melanoma and a colon carcinoma, was suppressed when cells were injected in admixture with BCG. Distant injection of BCG was ineffective. Mice with progressive growths had no detectable anti-human antibody, and rejection of cells and BCG failed to confer protection against subsequent tumour challenge. These studies indicate that human malignant cells are susceptible to local BCG-activated host responses, and that athymic mouse xenografts may be a useful model for assessing the response of human tumours to such agents.**

FOLLOWING THE WORK of Morton *et al.* (1970), it is well established that intralesional injection of *Bacillus Calmette–Guérin* (BCG) into cutaneous melanoma lesions in man may cause their regression (Goodnight & Morton, 1978). Other tumours, including those of breast (Smith *et al.*, 1973), bladder (Morales *et al.*, 1978) and prostate (Merrin *et al.*, 1975) have been shown to regress after local infiltration with BCG, and recently intrapleural BCG has been reported to prolong post-operative survival of Stage I lung cancer patients (McKneally *et al.*, 1976, 1978). While comparable studies with a wide range of experimental animal tumours have also shown their susceptibility to locally applied bacterial adjuvants, principally BCG and *C. parvum* (reviewed by Milas & Scott, 1978; Baldwin & Pimm, 1978), experimental techniques for studying the susceptibility of human tumours to this form of adjuvant contact suppression are clearly limited. However, in view of the current use of human tumour xenografts in mice for assessing their response to chemotherapeutic agents (Sonis *et al.*, 1977; UICC Technical Report, 1974; Povlsen & Jacobsen 1975; Houghton & Houghton, 1978), the present tests were carried out to examine their susceptibility to BCG. These studies were modelled on those previously carried out with rat tumour xenografts in congenitally athymic nude mice (Pimm & Baldwin, 1975, 1976).

### MATERIALS AND METHODS

*Tumour lines.*—Bladder carcinoma, Line T24, was obtained from Dr Michael Moore, Paterson Laboratories, Christie Hospital and Holt Radium Institute, Manchester.

Colon carcinoma, Line HT-29 was obtained from Dr L. M. Franks, Imperial Cancer Research Fund Laboratories, London.

Melanoma, Line Mel-S, was obtained from Dr C. Sorg, Universitäts-Hautklinik, Münster.

Lines were maintained by *in vitro* passage in Eagle's medium supplemented with 20% foetal calf serum.

*Bacillus Calmette–Guérin.*—Freeze-dried BCG vaccine (percutaneous) was supplied by Glaxo Laboratories, Greenford, Middlesex. On reconstitution this vaccine contains 50–250 $\times 10^6$ viable units in 3 mg moist wt/ampoule.

*Athymic mice.*—Athymic (CBA *nu/nu* or ONU *nu/nu*) mice and heterozygous mice (CBA nu/ +) were purchased from MRC Laboratory Animal Centre, Carshalton, Surrey. They were fed standard laboratory diet (Oxoid) and tapwater *ad libitum*, while housed in all-plastic cages with sawdust bedding in a Filter Rack Ventilation Cabinet (Anglia Laboratory Animals, Alconbury, Huntingdon, Cambridge).

*Antihuman serum.*—Heterozygous (*nu*/+) CBA mice received 2 i.p. injections at 14-day intervals of $10^6$ normal human lymphocytes or culture-derived T24 bladder carcinoma cells. They were bled from the heart 10 days after the second injection and sera stored at −20 °C.

*Indirect membrane-immunofluorescence tests.*—Mouse sera were tested for antibody against tumour cells by an indirect membrane-immunofluorescence test against viable cells in suspension, harvested from tissue culture or prepared by trypsin digestion of minced tissue taken from xenograft growths in athymic mice. $2\text{–}5 \times 10^6$ cells were incubated for 20 min at room temperature with 0·1 ml serum, washed ×4 in Medium 199, resuspended in 0·1 ml fluorescein-labelled goat anti-mouse globulin (Nordic Diagnostics, London, diluted 1/30) for 20 min, and finally washed ×4 and suspended in 1 : 1 (v/v) glycerol : phosphate-buffered saline (pH 7·2). Cells were examined with a Reichert fluorescence microscope and cells showing complete or partial membrane fluorescence scored as positively stained. Normal mouse serum (*nu*/*nu* or *nu*/+) was used as negative control and a fluorescence index (FI) calculated for each test serum as:

$$\text{FI} = \frac{\%\ \text{cells unstained by normal serum} - \%\ \text{cells unstained by test serum}}{\%\ \text{cells unstained by normal serum}}$$

*Experimental protocol.*—Cells were harvested from *in vitro* culture, washed and resuspended in Medium 199 or prepared from solid growths in athymic mice by digestion of minced tissue in 0·25% trypsin. Defined cell numbers were injected s.c. into groups of athymic mice alone or in admixture with BCG organisms. In one test, BCG was injected separately, by the i.p. route. Mice rejecting mixed inocula of tumour cells and BCG were in some cases challenged at a contralateral s.c. site with tumour cells alone.

### RESULTS

### *Growth and BCG suppression of tumours*

Groups of mice were injected s.c. with $1\text{–}2 \times 10^6$ human tumour cells, harvested from tissue culture or prepared by trypsin digestion of xenograft growths, alone or in admixture with BCG (Table I).

With all 3 tumours progressive growth occurred in the majority of mice receiving tumour cells alone. In contrast, admixture with BCG (0·5 mg moist wt) prevented tumour development in all animals (Table I). BCG injected at a distant, i.p. site failed to control growth of an s.c. chal-

TABLE I.—*BCG suppression of human tumour xenografts in athymic nude mice*

| | Mixed s.c. inoculum | | | | Challenge inoculum | | |
|---|---|---|---|---|---|---|---|
| | | | Tumour takes | | | Tumour takes | |
| Tumour | No. cells ($\times 10^6$) | BCG (mg) | Test | Control | No. cells ($\times 10^6$) | Test | Control |
| Melanoma | | | | | | | |
| Mel-S/TC* | 1 | 0·5 | 0/2 | 5/6 | | | |
| Colon carcinoma | | | | | | | |
| HT-29/TC/AM† | 1 | 0·5 | 0/2 | 2/2 | | | |
| | 1 | 0·5 | 0/3 | 2/2 | 1 | 3/3 | 3/3 |
| Bladder carcinoma | | | | | | | |
| T24/TC | 2 | 0·5 | 0/2 | 2/2 | | | |
| | 1 | 0·5 | 0/5 | 3/5 | | | |
| | 2 | 0·5 | 0/2 | 2/2 | 1 | 2/2 | 3/3 |
| | 1 | 0·5‡ | 3/3 | 3/3 | | | |

* TC—cells harvested from tissue culture.
† AM—cells prepared from xenograft growths in athymic mice.
‡ BCG injected i.p.

lenge. Groups of mice rejecting tumour cells and BCG were subsequently challenged s.c. in the opposite flank with cells alone of the same tumour (Table I). There was no resistance to this second challenge, tumour growing out in all mice, and in new control animals.

*Characteristics of xenograft growths*

Tumours developed as discrete encapsulated growths, with no macroscopically visible metastases. The melanoma Mel-S showed macroscopically visible pigmentation. To confirm that growths initiated in the mice from culture-derived tumour lines were indeed human, indirect membrane-immunofluorescence tests were carried out with mouse anti-human serum on viable cells brought into suspension (Table II). Cells from growths of all 3 tumour types reacted strongly with serum raised in heterozygous (*nu*/ +) mice against human peripheral lymphocytes. The lack of complete reactivity of serum against cells from digests of Mel-S growths (FI 0·95) could be a reflection of the presence of host (mouse) cells in the preparation, but the complete reactivity against HT29 and T24 does not imply that mouse stroma and blood vessels are not an integral part of these growths, rather that the technique of tumour digestion and cell handling may not be suitable for the recovery of such cells.

TABLE II.—*Immunofluorescence reactions (FI) of anti-human sera* against cells from athymic mouse xenografts*

| Tumour | FI against target cells derived from: Tissue culture | Athymic mouse |
|---|---|---|
| Melanoma-Mel-S | — | 0·95 |
| Colon carcinoma-HT29 | — | 1·00 |
| Bladder carcinoma-T24 | 1·00 | 1·00 |

* Raised in heterozygous *nu*/+ mice against human lymphocytes.

*Immunofluorescence tests with xenograft-bearing mouse sera*

Sera collected from 3 athymic mice bearing xenografts of the bladder carcinoma T24 were tested for anti-human antibody by the indirect membrane-immunofluorescence test against T24 cells prepared from growths in athymic mice or harvested from *in vitro* culture (Table III). None of the sera reacted against T24 cells (FI 0·00–0·01), although serum from heterozygous (*nu*/+) mice rejecting T24 cells reacted strongly (FI 0·79–1·00).

DISCUSSION

These studies demonstrate that cells of human tumours will produce progressive growth in congenitally athymic mice, and that this growth is prevented by BCG incorporated into the inoculum, not injected separately. Sere raised in heterozygous mice against normal human cells reacted with cells from xenografts, confirming the human characteristic of the cells.

In previous work, growth of rat tumour xenografts was similarly controlled by admixture with BCG or *C. parvum* (Pimm & Baldwin, 1975, 1976), although the suppression in athymic mice was in accordance with that seen in syngeneic recipients, so that carcinogen-induced sarcomas and hepatomas were readily controlled, while only slight suppression

TABLE III.—*Immunofluorescence reactions of sera from athymic mice bearing xenografts of human bladder carcinoma T24*

| Cells injected *in vitro* passage | Serum donor tumour: Age (days) | Mean diam. (cm) | Immunofluorescence reaction: Target cells | FI |
|---|---|---|---|---|
| T24/8 | 81 | 1·4 | T24-tissue culture | 0·00 |
| T24/8 | 81 | 1·4 | T24-athymic mouse | 0·00 |
| T24/8 | 74 | 1·7 | T24-athymic mouse | 0·01 |
| — | (*nu*/+) anti-T24 serum | | T24-tissue culture | 0·79–1·00 |

of a carcinogen-induced mammary carcinoma was achieved in either syngeneic rats or athymic mice.

There is considerable evidence, from work with syngeneically transplanted tumours and with athymic mouse xenografts, that tumour suppression by locally applied BCG is dependent upon local activation of host macrophages. Thus the response against rat tumours, both in syngeneic animals and athymic mice, is abrogated by depletion of host phagocytic cells with silica or carrageenan (Chassoux & Salomon, 1975; Hopper *et al.*, 1976; Moore & Nisbet, 1978; Keller, 1977). The indication from the present work is that locally activated host responses can similarly control growth of human malignant cells in an *in vivo* environment. This too is unlikely to involve systemic immunological responses, since distantly injected BCG was ineffective, and mice rejecting human malignant cells and BCG were not immune to a challenge with cells alone. Also immunofluorescence tests with sera from mice bearing xenografts failed to detect anti-human antibody. These observations parallel previous studies with rat tumour xenografts, where athymic mice rejecting cells and BCG were not immune to further challenge, and had no detectable anti-rat antibody (Pimm & Baldwin, 1975, 1976; Pimm, 1977).

This work was supported by a grant from the Cancer Research Campaign, and was carried out with the skilled technical assistance of Mrs A. P. Hopper, Mrs B. A. Jones and Mrs S. J. Wealthall. BCG was supplied by Glaxo Laboratories, Greenford, Middlesex, and the tissue culture lines were made available by Dr M. J. Embleton.

### REFERENCES

BALDWIN, R. W. & PIMM, M. V. (1978) BCG in tumour immunotherapy. *Adv. Cancer Res.* **28**, 91.

CHASSOUX, D. C. & SALOMON, J. C. (1975) Therapeutic effect of intratumoral injection of BCG and other substances in rats and mice. *Int. J. Cancer*, **16**, 515.

GOODNIGHT, J. E. & MORTON, D. L. (1978) Immunotherapy for malignant disease. *Ann. Rev. Med.*, **29**, 231.

HOPPER, D. G., PIMM, M. V. & BALDWIN, R. W. (1976) Silica abrogation of mycobacterial adjuvant contact suppression of tumour growth in rats and athymic mice. *Cancer Immunol. Immunother.*, **1**, 143.

HOUGHTON, P. J. & HOUGHTON, J. A. (1978) Evaluation of single-agent therapy in human colorectal tumour xenografts. *Br. J. Cancer*, **37**, 833.

KELLER, R. (1977) Abrogation of antitumour effects of *Corynebacterium parvum* and BCG by antimacrophage agents. *J. Natl Cancer Inst.*, **59**, 1751.

MCKNEALLY, M. F., MAVER, C. & KAUSEL, H. W. (1976) Regional immunotherapy of lung cancer with intrapleural BCG. *Lancet*, i, 377.

MCKNEALLY, M. F., MAVER, C. M. & KAUSEL, H. W. (1978) Regional immunotherapy of lung cancer using post operative intrapleural BCG. In: *Immunotherapy of Cancer: Present Status of Trials in Man*, Eds. W. D. Terry & D. Windhorst. New York: Raven Press, p. 161.

MERRIN, C., HAN, T., KLEIN, E., WAJSMAN, Z. & MURPHY, G. P. (1975) Immunotherapy of prostatic carcinoma with *Bacillus Calmette Guérin*. *Cancer Chemother. Rep.*, **59**, 157.

MILAS, L. & SCOTT, M. T. (1978) Antitumour activity of *Corynebacterium parvum*. *Adv. Cancer Res.*, **26**, 257.

MOORE, M. & NISBET, N. W. (1978) Abrogation of BCG-contact induced tumour inhibition by silica: implications for the mechanism of action. *Dev. Biol. Stand.*, **38**, 233.

MORALES, A., EIDINGER, D. R. & BRUCE, A. W. (1978) Adjuvant BCG immunotherapy in recurrent superficial bladder cancer. In: *Immunotherapy of Cancer: Present Status of Trials in Man*, Eds. W. D. Terry & D. Windhorst. New York: Raven Press, p. 225.

MORTON, D. L., EILBER, F. R., MALMGREN, R. A. & WOOD, W. C. (1970) Immunological factors which influence response to immunotherapy in malignant melanoma. *Surgery*, **68**, 158.

PIMM, M. V. (1977) Antigen expression on cells of rat tumour xenografts in athymic nude mice. *Br. J. Cancer*, **35**, 252.

PIMM, M. V. & BALDWIN, R. W.( 1975) BCG immunotherapy of rat tumours in athymic nude mice. *Nature*, **245**, 77.

PIMM, M. V. & BALDWIN, R. W. (1976) *C. parvum* suppression of rat tumours in athymic nude mice. *Br. J. Cancer*, **34**, 453.

POVLSEN, C. O. & JACOBSEN, G. K. (1975) Chemotherapy of a human malignant melanoma transplanted in the nude mouse. *Cancer Res.*, **35**, 2790.

SMITH, G. V., MORSE, P. A., DERAPS, J. D., RAJU, S. & HARDY, J. P. (1973) Immunotherapy of patients with cancer. *Surgery*, **74**, 59.

SONIS, S. T., FALCAO, R. P. & MACLENNAN, I. C. M. (1977) Assessment of drug sensitivity of human leukaemic myeloblasts. II. The toxic effects of cytosine arabinoside on $^{125}$IUdR-labelled human leukaemia myeloblasts in mice. *Br. J. Cancer*, **36**, 307.

UICC Technical Report (1974) Human tumor heterotransplantation in nude mice. In: *UICC Workshop on New Animal Models for Chemotherapy of Human Solid Tumors*, Eds. E. Mihich, D. J. R. Laurance & D. M. Eckhardt. UICC Tech. Rep. Series **15**. Geneva: International Union Against Cancer, p. 24.